# The novel risk score model for predicting the poor anticoagulation control in patients with atrial fibrillation taking warfarin

**DOI:** 10.2478/abm-2025-0013

**Published:** 2025-04-30

**Authors:** Komsing Methavigul, Rungroj Krittayaphong

**Affiliations:** 1 Department of Cardiology, Central Chest Institute of Thailand, Nonthaburi 11000, Thailand; 2 Department of Medicine, Faculty of Medicine, Siriraj Hospital, Mahidol University, Bangkok 10700, Thailand

**Keywords:** anticoagulant, atrial fibrillation, poor anticoagulation control, SAMe-TT_2_R_2_, time in therapeutic range

## Abstract

**Background:**

Previous trials have shown that the C-statistics of SAMe-TT_2_R_2_ score in the prediction of suboptimal time in therapeutic range (TTR) is very low.

**Objectives:**

To propose the novel risk score model for predicting the poor anticoagulation control in atrial fibrillation (AF) patients compared with the SAMe-TT_2_R_2_ score.

**Methods:**

We prospectively recruited AF patients from 27 hospitals between 2014 and 2017 in the COOL AF Thailand registry. The poor anticoagulation control was defined as TTR <65%. Multivariate logistic regression analysis was performed for the prediction of poor anticoagulation control. The novel risk score model was then generated. Receiver operating characteristic (ROC) curve analysis was performed to calculate the C-statistics and to compare between the novel risk score model and the SAMe-TT_2_R_2_ score. Net Reclassification Index (NRI) and Integrated Discrimination Index (IDI) were performed.

**Results:**

Of 3,461 patients, 2,233 patients taking warfarin having available TTR data were retrieved. There were 1,432 patients having poor anticoagulation control (TTR < 65%) and 801 patients having good anticoagulation control (TTR ≥ 65%). Symptomatic AF, diabetes, heart failure, and a history of bleeding were associated with increased risk while obesity, AF duration, and paroxysmal AF were associated with decreased risk of poor anticoagulation control. SHOB-D_2_AF score was created. The C-statistics of SHOB-D_2_AF score was greater than the SAMe-TT_2_R_2_ score (0.584 vs 0.506, *P* < 0.001). NRI of the SHOB-D_2_AF score was 17.82% compared with the SAMe-TT_2_R_2_ score.

**Conclusions:**

SHOB-D_2_AF score was the novel risk score which was better than the SAMe-TT_2_R_2_ score in predicting poor anticoagulation control.

Ischemic stroke is the most catastrophic sequelae in patients with atrial fibrillation (AF). Oral anticoagulants (OACs) are recommended by many international clinical practice guidelines in these patients, except in patients with very low-risk defined as CHA_2_DS_2_-VASc score of 0 in males and 1 in females [1–4]. Warfarin is the common OAC used in AF patients in Thailand [5]. Previous studies have shown that the time in therapeutic range (TTR) that targets the international normalized ratio (INR) between 2 and 3 of AF patients taking warfarin was 46.22%–53.17% [6, 7]. Moreover, previous trials have demonstrated that poor TTR is associated with adverse events including thromboembolism, bleeding, and/or mortality [8–10].

The SAMe-TT_2_R_2_ score was studied to predict the suboptimal TTR in patients taking vitamin K antagonists (VKAs) [7, 11–17]. However, the C-statistics of the SAMe-TT_2_R_2_ score in the prediction of suboptimal TTR is very low [17, 18]. Moreover, the “non-Caucasian” race that had 2 points on the SAMe-TT_2_R_2_ score limited the use of this score in Asians. To date, the European guidelines and the Asia-Pacific Heart Rhythm Society have recommended to consider VKA or non-vitamin K antagonist oral anticoagulants (NOACs) if the SAMe-TT_2_R_2_ score is 0–2 [1, 3]. However, a recent trial has demonstrated that AF patients taking warfarin have a significantly higher cardiovascular death or ischemic stroke/transient ischemic attack/systemic embolism or major bleeding compared with those taking NOACs, regardless of the SAMe-TT_2_R_2_ score [19]. This study aimed to propose the development of a novel risk score model for predicting the poor anticoagulation control in AF patients taking warfarin and compare the new risk score model with the SAMe-TT_2_R_2_ score.

## Methods

We prospectively recruited AF patients aged ≥18 years from 27 hospitals between 2014 and 2017 in the COhort of antithrombotic use and Optimal INR Level in patients with non-valvular AF in Thailand (COOL AF Thailand) registry. We excluded patients having prosthetic heart valve, rheumatic mitral valve disease, a history of recent ischemic stroke within 3 months, transient reversible cause of AF, predicted life expectancy <3 years, pregnancy, thrombocytopenia, and myeloproliferative diseases from this study.

We retrieved demographic and clinical data of AF patients taking warfarin stratified by anticoagulation control. We collected the baseline demographic data such as age, sex, body mass index (BMI), AF pattern and duration from AF diagnosis, AF-related symptoms, medical history including smoking and alcohol status, CHA_2_DS_2_-VASc score, HAS-BLED score, SAMe-TT_2_R_2_ score, left ventricular (LV) function, and concomitant medication. We recorded patient data at follow-up visits every 6 months. The poor anticoagulation control was defined as TTR <65%. We used the Rosendaal method for calculating TTR [20].

Each component of the SAMe-TT_2_R_2_ score was scored and recorded as S = female sex (1 point); A = age <60 years (1 point); Me = medical history >2 of the following: hypertension, diabetes, coronary artery disease (CAD)/myocardial infarction (MI), peripheral arterial disease (PAD), congestive heart failure (HF), previous stroke, pulmonary disease, and hepatic or renal disease (1 point); T = treatment (interacting drugs, e.g., amiodarone for rhythm control) (1 point); T_2_= tobacco use within 2 years (2 points); and R_2_ = non-Caucasian race (2 points).

This study protocol was approved by the Central Research Ethics Committee (CREC) (Certificate No.: COA-CREC003/2014). All participating patients provided written informed consent. We conducted this study protocol in compliance with the Declaration of Helsinki and the International Conference on Harmonization for Good Clinical Practice Guidelines (ICH-GCP).

### Statistical analysis

We used the descriptive statistics for analysis of demographic and clinical data. The categorical data were compared using Chi-square test and presented as numbers and percentage. The continuous data were compared using Student’s *t*-test and presented as mean and standard deviation (SD).

We used the univariate and multivariate analyses using logistic regression analysis in all demographic and clinical data for illustrating predictors associated with poor anticoagulation control. We generated the novel risk score model following predictors in multivariate analysis. The sensitivity, specificity, positive predictive value (PPV), and negative predictive value (NPV) for predicting poor anticoagulation control were calculated. Receiver operating characteristic (ROC) curve analysis was performed to calculate the C-statistics and to compare between the novel risk score model and SAMe-TT_2_R_2_ score. Sensitivity analysis was performed for comparison of the novel risk score model with the SAMe-TT_2_R_2_ score based on the predicted probability of each score system for the prediction of poor anticoagulant control calculated from the logistic regression analysis. Net Reclassification Index (NRI) and Integrated Discrimination Index (IDI) were performed based on the methods proposed in the previous publication to determine the influence of the novel risk score model on the reclassification of the study patients [21]. All analyses were performed by the SPSS statistical software version 18.0 (SPSS, Inc.) and R version 3.6.3 (www.r-project.org).

## Results

**Figure 1.**
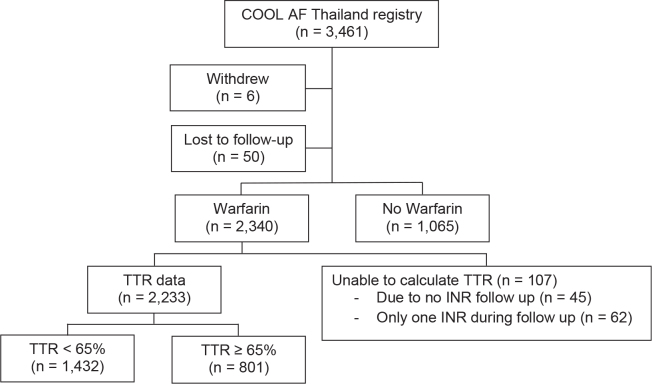
Flow diagram of study patients. INR, international normalized ratio; TTR, time in therapeutic range.

Of 3,461 patients, 2,233 patients taking warfarin having available TTR data were retrieved into this study. There were 1,432 patients having poor anticoagulation control (TTR <65%) and 801 patients having good anticoagulation control (TTR ≥ 65%) (Figure 1). The average age was 68.9 ± 10.6 years, and 44% were females. Baseline characteristics of the patients are shown in Table 1.

**Table 1.** Baseline characteristics of the study population

Variables	All patients (n = 2,233) n (%)	TTR ≥ 65% (n = 801) n (%)	TTR < 65% (n = 1,432) n (%)	*P*
**Age (years)**	68.9 ± 10.6	68.6 ± 10.6	69 ± 10.5	0.44
**Female sex**	980 (43.9)	351 (43.8)	629 (43.9)	0.96
**BMI (kg/m^2^)**	25.3 ± 4.8	25.5 ± 4.6	25.1 ± 4.9	0.08
**Duration from AF diagnosis (years)**	3.6 ± 4.4	3.8 ± 4.7	3.4 ± 4.2	0.04*
**AF pattern**				0.09
Paroxysmal AF	631 (28.3)	249 (31.1)	382 (26.7)	
Persistent AF	421 (18.9)	145 (18.1)	276 (19.3)	
Permanent AF	1,181 (52.9)	407 (50.8)	774 (54.1)	
**Symptomatic AF**	1,720 (77.0)	592 (73.9)	1,128 (78.8)	0.01*
**Medical history**				
Diabetes	610 (27.3)	184 (23)	426 (29.7)	<0.01*
Hypertension	1,641 (73.5)	574 (71.7)	1,067 (74.5)	0.14
Dyslipidemia	1,320 (59.1)	485 (60.5)	835 (58.3)	0.30
CAD	356 (15.9)	124 (15.5)	232 (16.2)	0.66
MI	130 (5.8)	41 (5.1)	89 (6.2)	0.29
PAD	29 (1.3)	12 (1.5)	17 (1.2)	0.53
Heart failure	628 (28.1)	192 (24)	436 (30.4)	<0.01*
Previous ischemic stroke/TIA	485 (21.7)	193 (24.1)	292 (20.4)	0.04*
Liver cirrhosis	48 (2.1)	16 (2.0)	32 (2.2)	0.77
Pulmonary disease	23 (1.0)	9 (1.1)	14 (1.0)	0.74
CKD	1,226 (54.9)	421 (52.6)	805 (56.2)	0.10
Dementia	20 (0.9)	8 (1.0)	12 (0.8)	0.70
Hyperthyroidism	87 (3.9)	29 (3.6)	58 (4.1)	0.62
Hypothyroidism	56 (2.5)	17 (2.1%)	39 (2.7)	0.38
Anemia	899 (40.3)	285 (35.6)	614 (42.9)	<0.01*
History of bleeding	241 (10.8)	73 (9.1)	168 (11.7)	0.06
**Smoking status**				0.80
Current smoking	51 (2.3)	17 (2.1)	34 (2.4)	
Ex-smoking	363 (16.3)	135 (16.9)	228 (15.9)	
**Alcohol use**	73 (3.3)	22 (2.7)	51 (3.6)	0.30
**CHA_2_DS_2_-VASc score**	3.4 ± 1.6	3.3 ± 1.6	3.4 ± 1.5	0.08
**HAS-BLED score**	1.6 ± 1.0	1.5 ± 1.0	1.7 ± 1.0	<0.01*
**LVEF (%)**	59.7 ± 14.2	60.5 ± 13.5	59.2 ± 14.5	0.04*
**TTR (%)**	53.6 ± 26.4	81.5 ± 11.2	38 ± 18.3	<0.01*
**Concomitant medications**				
Antiplatelet	277 (12.4)	86 (10.7)	191 (13.3)	0.07
NSAIDs	37 (1.7)	14 (1.7)	23 (1.6)	0.80
Amiodarone/dronedarone	104 (4.7)	37 (4.6)	67 (4.7)	0.95
Flecainide/propafenone	18 (0.8)	6 (0.7)	12 (0.8)	0.82
Digitalis	371 (16.6)	126 (15.7)	245 (17.1)	0.40

1 *P* < 0.05 indicates statistical significance (bold and asterisk).

1 Variables are shown as mean ± SD or number (%).

1 AF, atrial fibrillation; BMI, body mass index; CAD, coronary artery disease; CKD, chronic kidney disease; LVEF, left ventricular ejection fraction;

1 MI, myocardial infarction; NSAIDs, non-steroidal anti-inflammatory drugs; PAD, peripheral arterial disease; SD, standard deviation; TIA, transient ischemic attack; TTR, time in therapeutic range.

**Figure 2.**
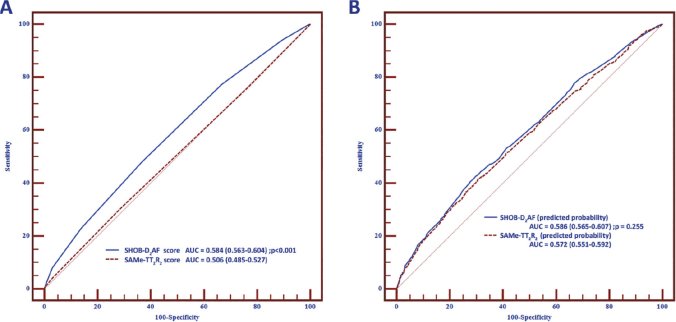
ROC curve illustrating discrimination performance between **(A)** SHOB-D_2_AF score and SAMe-TT_2_R_2_ score and **(B)** predicted probability derived from SHOB-D_2_AF score and SAMe-TT_2_R_2_ score. AF, atrial fibrillation; AUC, area under the curve; ROC, receiver operating characteristic.

**Figure 3.**
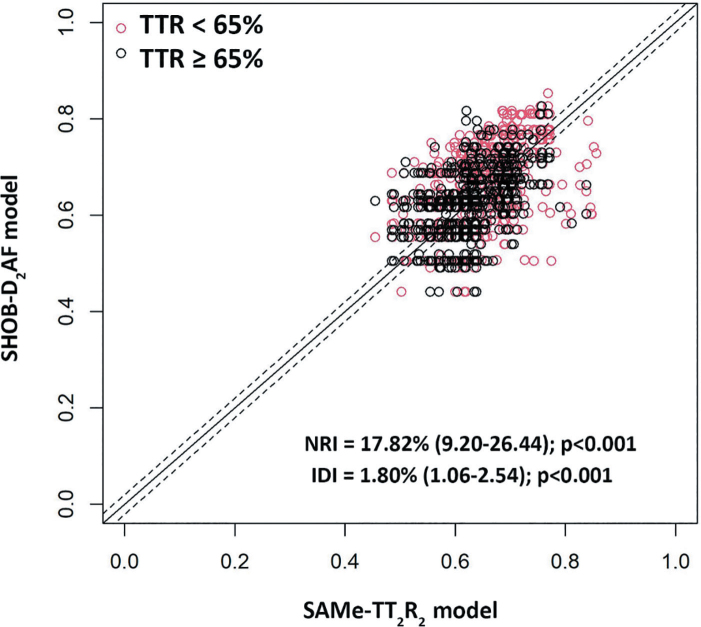
NRI of SHOB-D_2_AF compared with SAMe-TT_2_R_2_ model. AF, atrial fibrillation; IDI, Integrated Discrimination Index; NRI, Net Reclassification Index; TTR, time in therapeutic range.

Univariate and multivariate analysis showed that symptomatic AF, diabetes, HF, and a history of bleeding were associated with increased risk while obesity, AF duration, and paroxysmal AF were associated with decreased risk of poor anticoagulation control (Table 2). Diabetes, HF, and a history of bleeding were the most significant predictors of poor anticoagulation control. We scored each acronym of these predictors and generated the novel risk score model. The SHOB-D_2_AF score was created. The definition of this score is illustrated in Table 3. Mean TTR stratified by the SHOB-D_2_AF score and SAMe-TT_2_R_2_ score are shown in Table 4. ROC curve analysis showed that a SHOB-D_2_AF score of 4 or more could be used to predict poor anticoagulation control at the best cut-off point and predict better than the SAMe-TT_2_R_2_ score of 3 or more. The sensitivity, specificity, PPV, and NPV of the SHOB-D_2_AF score in predicting poor anticoagulation control were 47.9% (95% confidence interval [CI] 45.3%–50.5%), 63.3 (95% CI 59.9%–66.6%), 70.0% (95% CI 67.1%–72.8%), and 40.5% (95% CI 37.8%–43.2%), respectively, while the sensitivity, specificity, PPV, and NPV of the SAMe-TT_2_R_2_ score in predicting poor anticoagulation control were 76.3% (95% CI 74.0%–78.4%), 23.1 (95% CI 20.3%–26.2%), 64.0% (95% CI 61.7%–66.2%), and 35.2% (95% CI 31.3%–39.4%), respectively (Table 5). The SHOB-D_2_AF score significantly improved the discrimination performance compared with the SAMe-TT_2_R_2_ score (C-statistics 0.584 [0.563–0.604] vs 0.506 [0.485–0.527], *P* < 0.001) (Figure 2A). Sensitivity analysis was performed for comparison of the ROC curve from predicted probability derived from the SHOB-D_2_AF score and the SAMe-TT_2_R_2_ score. The C-statistics of predicted probability of the SHOB-D_2_AF score was comparable to the SAMe-TT_2_R_2_ score (0.586 [0.565–0.607] vs 0.572 [0.551–0.592], *P* = 0.255) (Figure 2B). The NRI was 17.82% (9.20%–26.44%), indicating that the SHOB-D_2_AF score performed better than the SAMe-TT_2_R_2_ score in predicting poor anticoagulation control (*P*< 0.001). The IDI was 1.80% (1.06%–2.54%) (*P* < 0.001) (Figure 3).

**Table 2.** Predictors of poor anticoagulation control

Predictors	Univariate analysis		Multivariate analysis	
	OR (95% CI)	*P*	OR (95% CI)	*P*
Age ≥75 (years)	1.10 (0.91–1.33)	0.32		
Female sex	1.00 (0.84–1.20)	0.96		
BMI ≥25 (kg/m^2^)	0.86 (0.73–1.03)	0.10	0.82 (0.68–0.98)	0.03*
Duration from AF diagnosis ≥2 years	0.76 (0.64–0.91)	<0.01*	0.73 (0.61–0.88)	<0.01*
AF pattern				
Paroxysmal AF	1.24 (1.03–1.50)	0.03*	0.78 (0.64–0.94)	0.01*
Persistent AF	1.08 (0.87–1.35)	0.497		
Permanent AF	1.14 (0.96–1.35)	0.14		
Symptomatic AF	1.31 (1.07–1.60)	0.01*	1.29 (1.05–1.59)	0.01*
Diabetes	1.42 (1.16–1.73)	<0.01*	1.49 (1.21–1.83)	<0.01*
Hypertension	1.16 (0.95–1.40)	0.14		
Dyslipidemia	0.91 (0.76–1.09)	0.30		
CAD	1.06 (0.83–1.34)	0.66		
MI	1.23 (0.84–1.80)	0.29		
PAD	0.79 (0.38–1.66)	0.54		
Heart failure	1.39 (1.14–1.69)	<0.01*	1.30 (1.07–1.59)	0.01*
Previous ischemic stroke/TIA	0.81 (0.66–0.99)	0.04*		
Liver cirrhosis	1.12 (0.61–2.06)	0.71		
Pulmonary disease	0.87 (0.37–2.02)	0.74		
CKD	1.16 (0.97–1.38)	0.10		
Dementia	0.84 (0.34–2.06)	0.70		
Hyperthyroidism	1.12 (0.71–1.77)	0.62		
Hypothyroidism	1.29 (0.73–2.30)	0.39		
Anemia	1.36 (1.14–1.63)	<0.01*		
History of bleeding	1.33 (0.99–1.77)	0.06	1.36 (1.01–1.83)	0.04*
Smoking status	0.96 (0.77–1.19)	0.69		
Alcohol use	1.31 (0.79–2.17)	0.30		
LVEF < 50%	1.28 (1.02–1.60)	0.03*		
Antiplatelet	1.28 (0.98–1.68)	0.07		
NSAIDs	0.92 (0.47–1.79)	0.80		
Amiodarone/dronedarone	1.01 (0.67–1.53)	0.95		
Flecainide/propafenone	1.12 (0.42–3.00)	0.82		
Digitalis	1.11 (0.87–1.40)	0.40		

1 *P* < 0.05 indicates statistical significance (bold and asterisk).

1 AF, atrial fibrillation; BMI, body mass index; CAD, coronary artery disease; CI, confidence interval; CKD, chronic kidney disease; LVEF; left ventricular ejection fraction; MI, myocardial infarction; NSAIDs, non-steroidal anti-inflammatory drugs; OR, odds ratio; PAD, peripheral arterial disease; TIA, transient ischemic attack.

**Table 3.** Definition of SHOB-D_2_AF score

Acronym	Description	Coefficient	Points
S	Symptomatic AF	0.258021	1
H	Heart failure	0.264288	1
O	Omit obesity (BMI < 25 kg/m^2^)	0.200332	1
B	Bleeding history	0.306595	1
D_2_	Diabetes	0.396291	2
AF	Duration from AF diagnosis <2 years	0.313393	1
	Non-paroxysmal AF	0.255041	1
	Constant	0.531131	
Maximum points			8

1 AF, atrial fibrillation; BMI, body mass index.

**Table 4.** Mean TTR stratified by SHOB-D_2_AF score and SAMe-TT_2_R_2_ score

Risk scoring system	No. of patients	Mean TTR ± SD
SHOB-D_2_AF score		
0	10	71.9 ± 28.2
1	160	59.0 ± 27.8
2	423	57.9 ± 25.4
3	660	55.2 ± 25.6
4	544	51.8 ± 26.9
5	297	48.5 ± 25.8
6	106	43.1 ± 25.3
7	32	40.9 ± 23.5
8	1	47.4 ± 0.0
**Total**	**2,233**	**53.6 ± 26.4**
SAMe-TT_2_R_2_ score		
2	526	53.8 ± 25.4
3	1,041	53.9 ± 27.2
4	583	53.2 ± 25.8
5	75	50.8 ± 26.0
6	8	45.7 ± 24.1
**Total**	**2,233**	**53.6 ± 26.4**

1 SD, standard deviation; TTR, time in therapeutic range.

**Table 5.** The sensitivity, specificity, predictive value, and AUC of the risk score system in predicting poor anticoagulation control

Risk scoring system	Sensitivity (95% CI)	Specificity (95% CI)	PPV (95%CI)	NPV (95% CI)	AUC (95% CI)
SHOB-D_2_AF score ≥4	47.9 (45.3–50.5)	63.3 (59.9–66.6)	70.0 (67.1–72.8)	40.5 (37.8–43.2)	0.584 (0.563–0.604)
SAMe-TT_2_R_2_ score ≥3	76.3 (74.0–78.4)	23.1 (20.3–26.2)	64.0 (61.7–66.2)	35.2 (31.3–39.4)	0.506 (0.485–0.527)

1 AUC, area under the curve; CI, confidence interval; NPV, negative predictive value; PPV, positive predictive value.

## Discussion

To our knowledge, high TTR is the most important marker to consider warfarin as an option for OAC in AF patients. Additionally, poor TTR has been associated with adverse outcomes in these patients [8–10]. The SHOB-D_2_AF score is the novel risk score model for predicting poor anticoagulation control in AF patients taking warfarin. The TTR decreased following the increased scores of these risk score models. Of note, the SHOB-D_2_AF score could be used to predict poor TTR better than the SAMe-TT_2_R_2_ score in this cohort study in terms of area under the curve (AUC) and NRI.

Previous trials have shown that the SAMe-TT_2_R_2_ score could predict the poor anticoagulation control in AF patients taking VKAs [7, 11–17]. However, those trials demonstrated that there were variable results of suboptimal INR prediction in terms of AUC from 0.54 to 0.70 [7, 11, 12, 16, 17]. In Thailand, previous SAMe-TT_2_R_2_ score trials have shown that AUC was 0.54–0.59, respectively [7, 10, 17]. Nevertheless, recent European guidelines recommend to choose VKAs or NOACs for stroke prevention stratified by the SAMe-TT_2_R_2_ score [1]. A previous study revealed that the SAMe-TT_2_R_2_ score could not be used to choose VKAs or NOACs in AF patients taking warfarin [19].

The SAMe-TT_2_R_2_ score having lower than expected accuracy for prediction of poor anticoagulation control may be from the magnitude of predictability in each parameter in this risk score. For example, there are many illnesses in medical history such as hypertension, diabetes, CAD/MI, PAD, congestive HF, previous stroke, pulmonary disease, and hepatic or renal disease. Each medical illness has a different predictability for poor anticoagulation control, such as congestive HF may have stronger predictability than hypertension or CAD/MI. This study showed that only diabetes and HF were the predictors of poor anticoagulation control, while hypertension, CAD/MI, PAD, previous stroke, CKD, and liver cirrhosis were not the predictors. Moreover, other risk predictors of poor anticoagulation control did not mention in the SAMe-TT_2_R_2_ score symptoms such as BMI, duration from AF diagnosis, pattern of AF, a history of bleeding, or AF-related symptoms. This trial demonstrated that these risk factors could predict poor anticoagulation control as well. Besides, the “non-Caucasian” race item in the SAMe-TT_2_R_2_ score makes this scoring system not suitable to be used in the Asian population. Finally, this study showed that the SHOB-D_2_AF score had superior PPV with comparable NPV and significantly improved the prediction of poor anticoagulation control in terms of AUC and NRI compared with the SAMe-TT_2_R_2_ score. In clinical practice, AF patients with a SHOB-D_2_AF score ≥4 should be recommended to choose NOACs over VKAs, while those patients with a SHOB-D_2_AF of <4 should be recommended to initiate VKAs first as well.

However, this study has several limitations. First, there were small patients having some risk predictors for poor anticoagulation control such as PAD, liver cirrhosis, dementia, hypothyroidism, hyperthyroidism, and amiodarone. However, our risk score model still had more predictors and could predict better poor anticoagulation than the SAMe-TT_2_R_2_ score. Second, this study was an observational study leading to selection bias. Nevertheless, we analyzed all predictors in univariate and multivariate analysis. However, our risk score model needs validation in another dataset of Thai patients. Finally, our patients were Thai, leading to limit generalizability. The non-Caucasian race is the predictor of poor anticoagulation control in the SAMe-TT_2_R_2_ score, but this study has had no other race included in our analysis. Our risk score model will need to conduct studies on other races in the future.

## Conclusion

The SHOB-D_2_AF score was the novel risk score model that was better than the SAMe-TT_2_R_2_ score in predicting poor anticoagulation control.
